# Exploring the Therapeutic Potential of Ginseng in Diabetic Retinopathy: A Network Pharmacology and Molecular Docking Study

**DOI:** 10.1155/abb/5493390

**Published:** 2025-10-28

**Authors:** Yuanchao Fu, Chengzhi Liu, Guzhi Xu, Chuanlie Zhou

**Affiliations:** ^1^ Department of Rehabilitation Medicine, Affiliated Dalian Friendship Hospital of Dalian Medical University, Dalian, China; ^2^ The First Affiliated Hospital of Dalian Medical University, Dalian, China, dlmedu.edu.cn

**Keywords:** AGE-RAGE signaling pathway, diabetic retinopathy, ginseng, molecular docking, network pharmacology, TCMSP

## Abstract

Diabetic retinopathy (DR), a major cause of vision impairment, results from hyperglycemia‐induced retinal microvascular damage. Current therapies mainly address symptoms rather than underlying mechanisms. Ginseng, a traditional medicinal herb with antioxidant and anti‐inflammatory properties, may offer therapeutic benefits for DR. This study employed network pharmacology to identify bioactive compounds in ginseng and their potential molecular targets associated with DR. Gene Ontology (GO) and Kyoto Encyclopedia of Genes and Genomes (KEGG) analyses revealed significant enrichment in inflammation‐ and oxidative stress‐related pathways, particularly the advanced glycation end product (AGE)‐RAGE signaling pathway. A protein–protein interaction (PPI) network identified key targets, and molecular docking and dynamics simulations confirmed strong binding affinities between ginseng compounds and DR‐related proteins. These findings suggest ginseng may modulate critical pathways involved in DR progression, offering potential as a natural therapeutic agent.

## 1. Introduction

Diabetic retinopathy (DR) is a major complication of diabetes mellitus (DM) and the leading cause of vision loss in the working‐age population, significantly impacting quality of life [1]. The prevalence of DR is increasing annually, leading to a growing demand for eye care and treatment [2]. A meta‐analysis estimated that the number of DR patients was ~103.12 million in 2020, with projections indicating this number could increase to 160.50 million by 2045 [3]. The pathological process of DR is complex and involves damage to retinal blood vessels and nerves. The clinical diagnosis of DR mainly depends on discovering vascular lesions detected by fundus examination [4]. DR can be divided into two main pathological stages: non‐proliferative DR (NPDR) and proliferative DR (PDR) [9]; the latter is characterized by the formation of neovascularization followed by retinal and vitreous hemorrhage, and causes the rapid decrease of visual acuity [5, 6]. Due to the inconspicuous clinical manifestations in NPDR, current therapies primarily comprise photocoagulation, vitrectomy, and intraocular injection of anti‐vascular endothelial growth factor (anti‐VEGF) or glucocorticoids, with a main focus on the advanced stage of PDR, where treatment effects are often poor [4, 7]. Therefore, exploring and implementing novel therapeutic approaches is crucial for managing and treating DR patients [8].

Ginseng has been used for centuries in traditional Chinese medicine, with many ancient Chinese medical texts documenting its wide‐ranging therapeutic and health‐promoting properties [9]. Its pharmacological effects have been well‐documented over centuries, contributing to its esteemed status in Chinese herbal medicine. As a result of its notable pharmacokinetics, a growing body of research has focused on ginseng, exploring its potential applications and health benefits. This increasing scientific interest underscores ginseng’s enduring relevance and potential in modern medicine [10, 11]. Previous studies have demonstrated that ginseng can mitigate diabetic complications across multiple organs, including the heart and retina [12]. In experimental models of both type 1 and type 2 diabetes, administration of North American ginseng significantly improved systemic metabolic dysregulation, reduced oxidative stress, and inhibited diabetes‐induced upregulation of extracellular matrix proteins and vasoactive factors. In the retina, ginseng prevented oxidative stress–mediated biochemical and vascular alterations that contribute to the development of DR [13]. Collectively, these findings suggest that ginseng may protect against diabetes‐induced tissue injury; however, its precise mechanisms of action remain unclear and need further investigation.

Traditional Chinese medicine is characterized by its use of multiple components, targets, and pathways to treat diseases, which can complicate exploring underlying mechanisms. Network pharmacology has emerged as a novel bioinformatics strategy that enables the study of interactions between drug components and biological systems by mapping drug‐target‐disease networks, thereby addressing this complexity. Numerous studies have shown that combining network pharmacology with molecular docking methods or kinetic simulations can effectively predict the components, targets, signaling pathways, and mechanisms involved in herbal treatments for various diseases [14, 15]. This approach saves time and resources and aids in identifying active ingredients and understanding their mechanisms of action [16].

**Figure 1:**
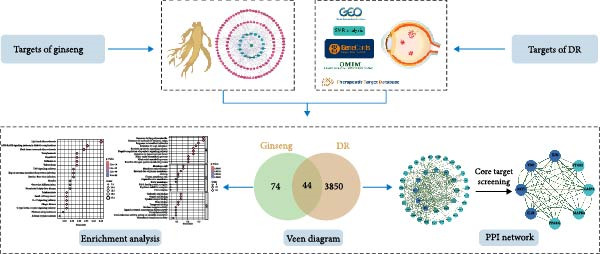
Flowchart outlining the entire analysis process.

Building on these benefits, our study aims to utilize these approaches to identify the therapeutic components of ginseng for treating DR and to explore the mechanisms through which ginseng exerts its effects (Figure 1). By employing network pharmacology and related techniques, we hope to uncover new insights into the role of ginseng in managing DR, potentially leading to more effective treatment strategies.

## 2. Methods

### 2.1. Screening of Pharmacologically Active Ingredients in Ginseng

To identify the pharmacologically active ingredients of ginseng, we utilized the Traditional Chinese Medicine Systems Pharmacology Database and Analysis Platform (TCMSP, http://lsp.nwu.edu.cn/index.php) [17]. Using “ginseng” as the keyword, we retrieved comprehensive information on the herb’s active components. The TCMSP database provides detailed data on the absorption, distribution, metabolism, and excretion (ADME) properties of traditional Chinese medicine ingredients, including metrics such as oral bioavailability (OB), drug‐likeness (DL), and half‐life (HL). For this study, we focused on selecting active ingredients with OB ≥ 30% and DL ≥ 0.18 as our primary research subjects. These criteria ensure the selected compounds have suitable pharmacokinetic profiles for therapeutic use. Additionally, we obtained the three‐dimensional (3D) chemical structures of the chosen ginsenosides in mol2 format from the TCMSP database, which will be used for further analysis, such as molecular docking and network pharmacology studies.

### 2.2. Screening of the Potential Target Genes Related to DR

We explored genes associated with DR pathogenesis using transcriptome data, focusing on differentially expressed genes (DEGs) as potential contributors to the disease. The raw data for DR were obtained from the NCBI Gene Expression Omnibus (GEO) database (https://www.ncbi.nlm.nih.gov/geo/), specifically dataset GSE60436, which includes three healthy retinal samples and six retinal fibrovascular membrane samples from patients with PDR. Differential gene expression analysis was performed using GEO2R, with DEGs identified based on the screening criteria of an adjusted *p*‐value < 0.05 and |LogFC| > 1 [18].

Summary data‐based Mendelian randomization (SMR) is a statistical method used to evaluate the causal relationship between exposure (such as genetic variation or gene expression) and outcomes (such as disease or trait) using aggregated level data from genome‐wide association studies (GWAS) and expression quantitative trait loci (eQTL) studies. This method utilizes the principle of Mendelian randomization, which uses genetic variation as an instrumental variable (IV) to infer causal relationships, assuming that these variations are related to exposure but unrelated to confounding factors. The SMR method is particularly suitable for integrating GWAS and eQTL data to identify genes whose expression levels may be causally associated with specific traits or diseases. In this SMR study, cis‐eQTL was selected as the IV for gene expression, and the cis‐eQTL data and corresponding allele frequencies were recorded in eQTLGen (https://www.eqtlgen.org/cis-eqtls.html) [19]. The DR GWAS data comes from the FinnGen database (r9, https://r9.finngen.fi/ GWAS ID: Finngen_R9_DN_RETINOPATHY_EXMORE). SMR software (version 1.03, https://yanglab.westlake.edu.cn/software/smr/#Overview) was used for SMR analysis and the heterogeneity in dependent instruments (HEIDI) test. Genes with an odds ratio (OR) > 1 and passing the HEIDI test are considered DR‐related genes [20].

To identify more potential therapeutic targets for DR, we searched the online databases using the keyword “diabetic retinopathy” as follows: Genecards database (https://www.genecards.org/) [21], Online Mendelian Inheritance in Man (OMIM) database (https://www.omim.org/) [22], and Therapeutic Target Database (TTD, http://db.idrblab.net/ttd/) [23]. Microsoft Excel was utilized to remove duplicate items and identify DR‐related targets. Venn diagrams were then used to visually demonstrate the overlap of targets obtained by each method.

### 2.3. Gene Ontology (GO) and Kyoto Encyclopedia of Genes and Genomes Pathway (KEGG) Enrichment Analysis

To investigate the function of the targets and their roles in signal transduction, we utilized Bioconductor R software to identify enriched GO terms and KEGG pathways. The GO terms were categorized into three main criteria: molecular function, cellular component, and biological process. Only GO and KEGG terms with a corrected *p*‐value of < 0.05 (using Bonferroni adjustment) were retained and analyzed. The results were visualized using R software, and the signaling pathway diagram was sourced from the KEGG database (https://www.kegg.jp/kegg/kegg1.html) [24].

### 2.4. The Construction of a Protein–Protein Interaction (PPI) Network and Identification of Core Targets

To investigate the potential interactions between targets, we utilized the STRING platform (https://string-db.org) to construct a PPI network, setting the parameters to “*Homo sapiens*” with a confidence score of 0.700 to ensure reliable results. The PPI network was then visualized using Cytoscape 3.7.0 software (https://cytoscape.org/). The “CentiScaPe” plugin in Cytoscape 3.7.0 was employed to calculate the topological parameters of the network. Degree centrality (DC) was used as the primary measure, while betweenness centrality (BC) and closeness centrality (CC) served as secondary metrics to identify core targets. This approach ultimately revealed the key target for ginseng in treating DR.

### 2.5. Molecular Docking

Molecular docking is a powerful tool used in drug discovery to predict and design new drugs by simulating the binding patterns between drug ligands and target proteins in 3D structures [25]. This method helps predict potential docking configurations and binding affinities, aiding in drug discovery. In this study, we performed molecular docking between key targets from the PPI network and the active ingredients in ginseng that act on these targets. The molecular 3D structures of active ingredients and key targets were obtained from the TCMSP database and RCSB PDB (http://www.rcsb.org/), respectively. Docking was performed using AutoDock Tools 1.5.7, and the results were visualized using PyMol 2.5.7 [26, 27]. Specifically, prior to molecular docking, receptor proteins were pre‐processed using AutoDock Tools 1.5.7 by removing crystallographic water molecules, adding polar hydrogens, and assigning Gasteiger charges. Ligand structures were energy‐minimized and converted into the required PDBQT format with defined torsional flexibility. Docking was carried out using the Lamarckian Genetic Algorithm implemented in AutoDock, and the binding energy (kcal/mol) was used to rank the conformations. The best docking pose for each ligand–receptor complex was selected based on the lowest binding free energy and favorable interaction geometry. The docking results were visualized and analyzed using PyMol 2.5.7.

### 2.6. Molecular Dynamics (MD) Simulations

MD simulations were performed using GROMACS 2023.2. The protein–ligand complex structure was uploaded to the Solution Builder module of CHARMM‐GUI, where a solvent box was constructed with a minimum distance of 10 Å from the outermost atoms of the complex. The system was solvated with TIP3 water molecules, and Na^+^ and Cl^−^ ions were added to neutralize the system and maintain an ionic concentration of 0.15 mol/L. The temperature was set to 303.15 K, and periodic boundary conditions were applied. The particle mesh Ewald (PME)‐FFT method was used to automatically generate grid information for long‐range electrostatics. CHARMM36m force field parameters were assigned to all atoms in the system to generate the topology files. Energy minimization was carried out using the steepest descent algorithm for a maximum of 5000 steps. The PME method was used for long‐range electrostatics. The energy minimization terminated when the maximum force (emtol) on any atom was less than 100 kJ/mol·nm. After energy minimization, NPT equilibration was performed for 125 ps under constant volume and a gradual heating scheme. The final MD simulation was conducted for 100 ns with a time step of 2 fs. Visualization and analysis were performed using DuIvyTools 0.6.0. Once the system (particularly the ligand) reached stability, the binding free energy between the protein and ligand was calculated using gmx_MMPBSA, based on the following equations:

∆TOTAL=∆GGAS+∆GSOLV,

∆GGAS=∆VDWAALS+∆EEL,

∆GSOLV=∆EGB+∆ESURF.

## 3. Results

### 3.1. Pharmacologically Active Ingredients in Ginseng

Using the TCMSP database, we extracted ginseng’s active ingredients and targets. After removing duplicate compounds, a total of 22 active ingredients were identified under the conditions of DL ≥ 0.18 and OB ≥ 30%. The specific details of these ingredients are provided in Table 1. Additionally, these 22 active ingredients correspond to 117 related targets (Supporting Information 1: Table S1), and the targets corresponding to each active ingredient are obtained. Cytoscape was used to visualize these active ingredients and their corresponding hypothesized targets to understand the relationships better (Figure 2A). In addition, we also conducted functional enrichment analysis on the targets of ginseng, as shown in Figure 2B,C.

Figure 2Screening and visualization of ginseng targets. (A) Network analysis visualizes the interaction between ginseng targets (green nodes) and associated proteins (pink nodes). The connections (edges) represent predicted interactions between these elements, illustrating the complexity of ginseng’s potential mechanisms of action. (B) GO enrichment analysis highlights the biological processes, molecular functions, and cellular components most significantly associated with the identified ginseng targets. The size of the dots represents the count of genes, and the color gradient reflects the *p*‐value, with darker colors indicating higher significance. (C) KEGG pathway analysis shows the top pathways enriched by the ginseng targets, with dot size representing gene count and color intensity indicating significance levels.

**Figure:**
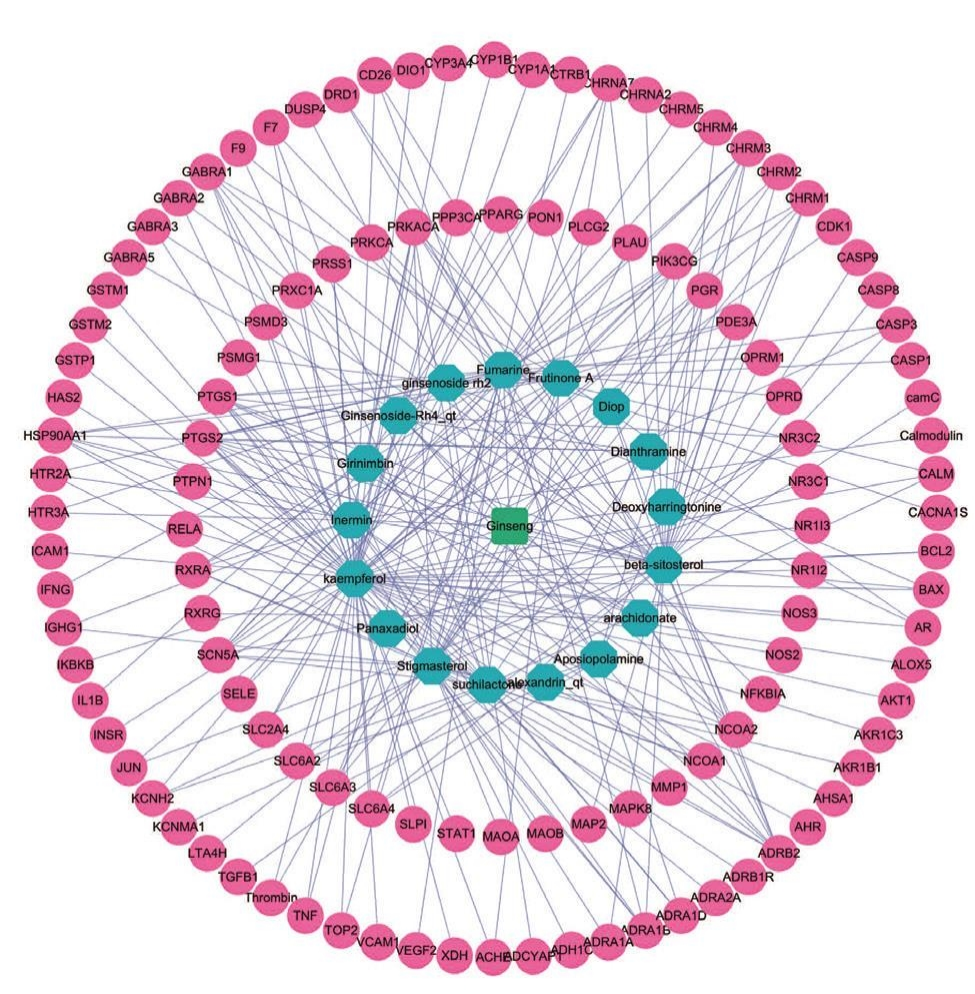
(A)

**Figure:**
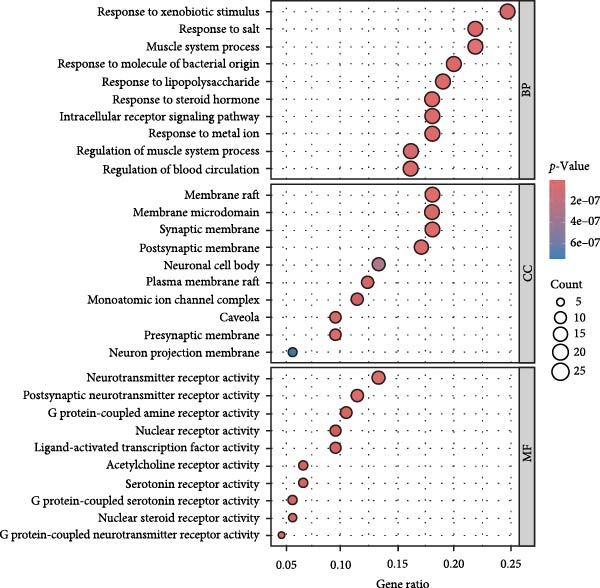
(B)

**Figure:**
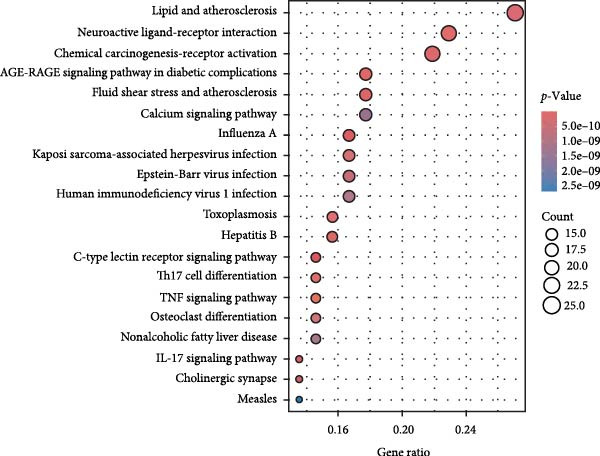
(C)

**Table 1:** Information on active compounds in ginseng and their molecular targets.

Mol ID	Molecule name	OB (%)	DL	Targets
MOL002879	Diop	43.59	0.39	3
MOL000449	Stigmasterol	43.83	0.76	31
MOL000358	Beta‐sitosterol	36.91	0.75	38
MOL003648	Inermin	65.83	0.54	18
MOL000422	Kaempferol	41.88	0.24	61
MOL005308	Aposiopolamine	66.65	0.22	8
MOL005317	Deoxyharringtonine	39.27	0.81	2
MOL005318	Dianthramine	40.45	0.2	3
MOL005320	Arachidonate	45.57	0.2	4
MOL005321	Frutinone A	65.9	0.34	16
MOL005344	Ginsenoside rh2	36.32	0.56	12
MOL005348	Ginsenoside‐Rh4_qt	31.11	0.78	2
MOL005356	Girinimbin	61.22	0.31	10
MOL005376	Panaxadiol	33.09	0.79	1
MOL005384	Suchilactone	57.52	0.56	16
MOL005399	Alexandrin_qt	36.91	0.75	1
MOL000787	Fumarine	59.26	0.83	28

### 3.2. Potential Target Genes Related to DR

We utilized multiple data sources to comprehensively identify DR‐related targets, including transcriptome sequencing, GWAS data, and public databases. Differential expression analysis of the GSE60436 dataset yielded 2642 DR‐related targets. Additionally, SMR analysis provided specific information on 64 targets. From public databases, we identified 1225 targets from GeneCards, 233 from OMIM, and 7 from TTD, as illustrated in Figure 3A,B. After integrating and deduplicating the data, we obtained 3894 unique DR‐related targets (Supporting Information 2: Table S2). Furthermore, functional enrichment analysis was performed on these targets (Figure 3C,D).

Figure 3Identification and functional enrichment of DR‐related targets. (A) Venn diagram displaying the overlap of DR‐related targets obtained from different sources: transcriptome sequencing data, SMR analysis, GeneCards, OMIM, and TTD databases. The numbers in each section represent the unique and shared targets among these datasets. (B) A bar chart shows the number of DR‐related targets identified from each source, highlighting the contribution of each dataset to the overall pool of targets. (C, D) GO and KEGG analysis of DR‐related targets.

**Figure:**
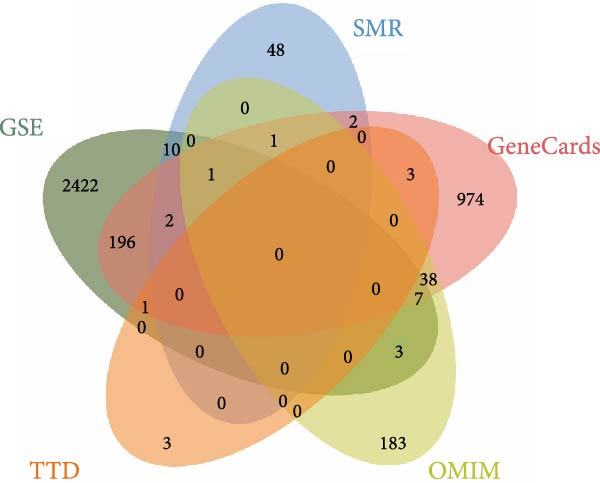
(A)

**Figure:**
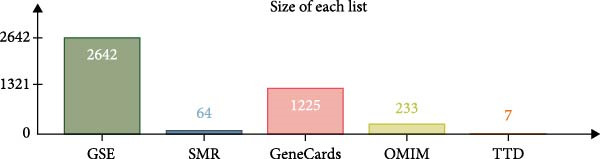
(B)

**Figure:**
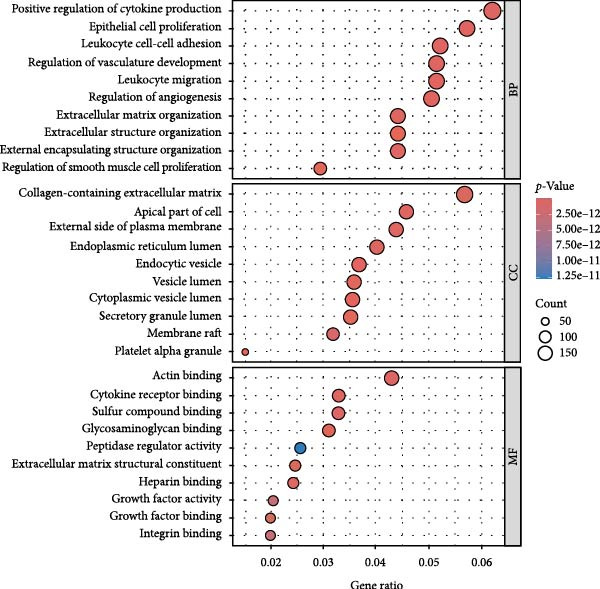
(C)

**Figure:**
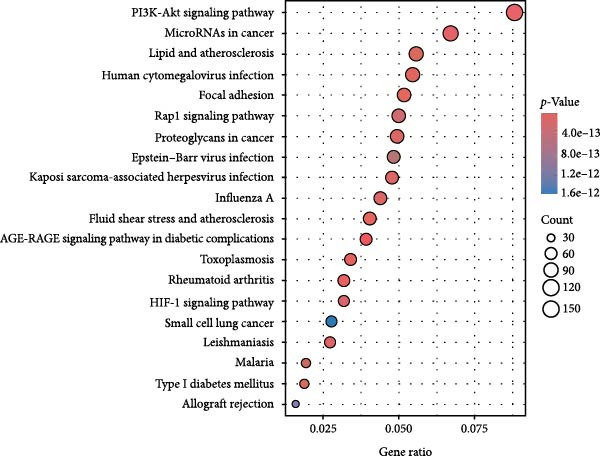
(D)

### 3.3. The Identification of the Shared Targets and Functional Enrichment Analysis

**Figure 5:**
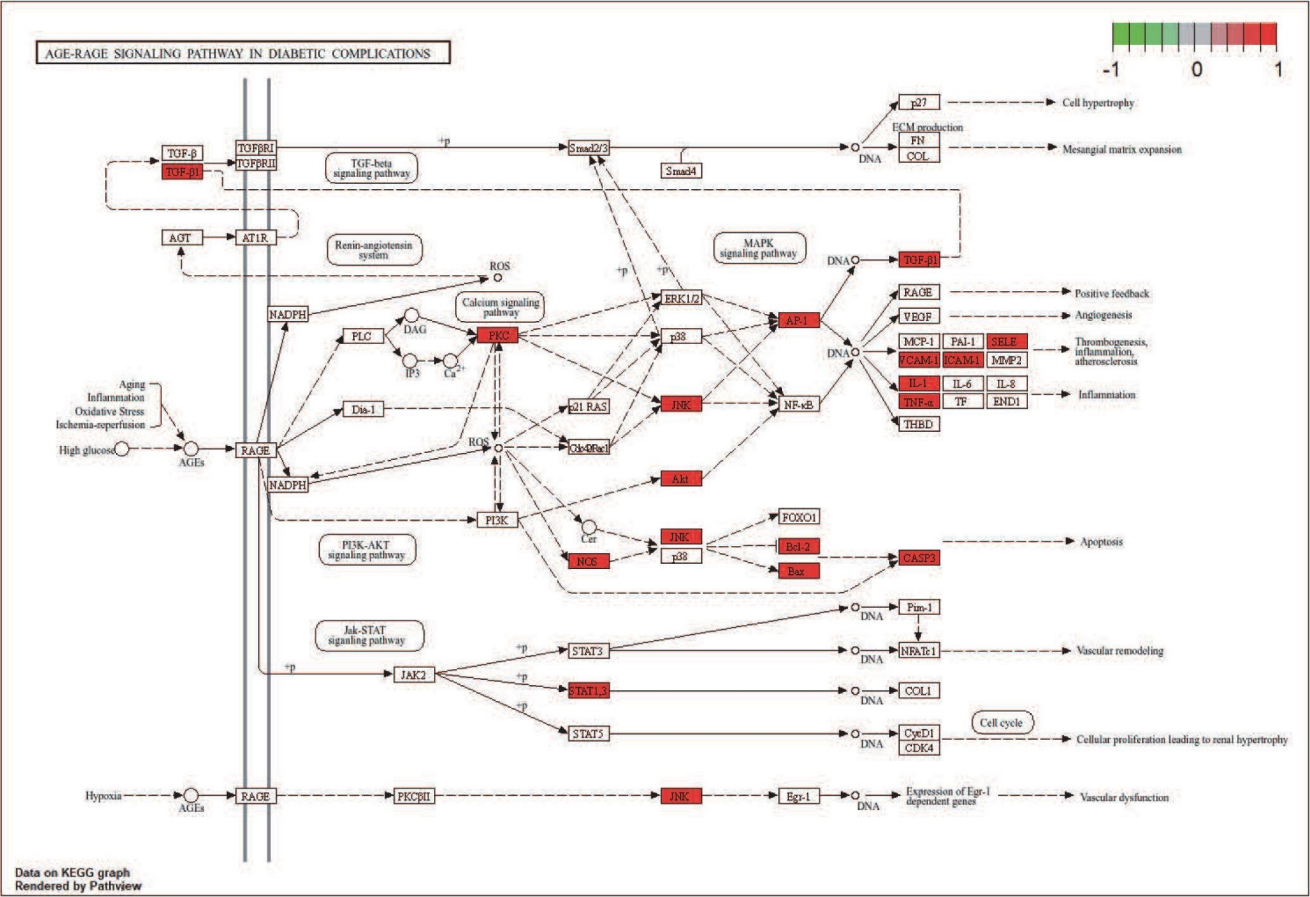
Detailed pathway map of the AGE‐RAGE signaling pathway in diabetic complications, with key components marked (KEGG ID: hsa04933).

We matched 117 potential ginseng‐related targets with 2642 potential DR‐related targets, identifying 44 common targets (Figure 4A). The GO analysis revealed several enriched biological processes including responses to lipopolysaccharide, bacterial molecules, xenobiotic stimuli, toxic substances, tumor necrosis factor (TNF), apoptotic signaling pathways, nitric oxide biosynthetic processes, and reactive nitrogen species metabolic processes. Cellular component highlighted associations with membrane rafts, membrane microdomains, the external side of the plasma membrane, caveolae, plasma membrane rafts, organelle outer membranes, Bcl‐2 family protein complexes, peptidase inhibitor complexes, and pore complexes. The molecular function terms included carboxylic acid binding, organic acid binding, cytokine receptor binding, heme binding, tetrapyrrole binding, nuclear receptor activity, ligand‐activated transcription factor activity, peroxidase activity, oxidoreductase activity acting on peroxide as an acceptor, and glutathione transferase activity (Figure 4B). KEGG pathway enrichment analysis highlighted several key pathways, including lipid metabolism and atherosclerosis, the advanced glycation end product (AGE)‐RAGE signaling pathway in diabetic complications, fluid shear stress and atherosclerosis, toxoplasmosis, and hepatitis B, among others (Figure 4C,D). The AGE‐RAGE signaling pathway plays a crucial role in the pathogenesis of diabetes and its associated complications. We visualized the molecules involved in the “AGE‐RAGE signaling pathway in diabetic complications” (hsa04933) across all targets (Figure 5).

Figure 4Identification and functional analysis of targets associated with ginseng and DR. (A) Venn diagram showing the overlap of targets between ginseng and DR, with 44 common targets identified. (B) GO enrichment analysis of the 44 common targets, highlighting biological processes and molecular functions significantly associated with these targets. (C) KEGG pathway enrichment analysis demonstrates key pathways in which the common targets are involved. (D) A network of common targets is mapped to the enriched pathways, illustrating the interconnectedness and potential mechanisms of action.

**Figure:**
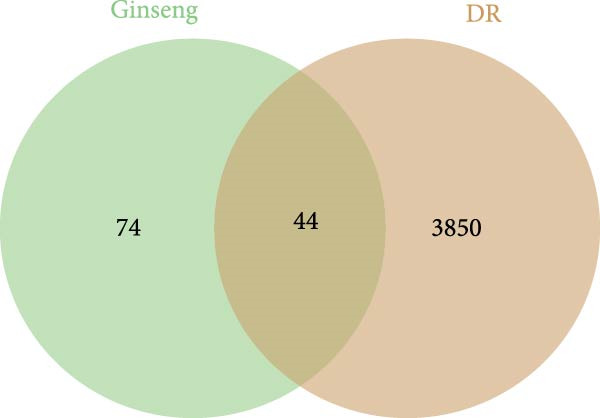
(A)

**Figure:**
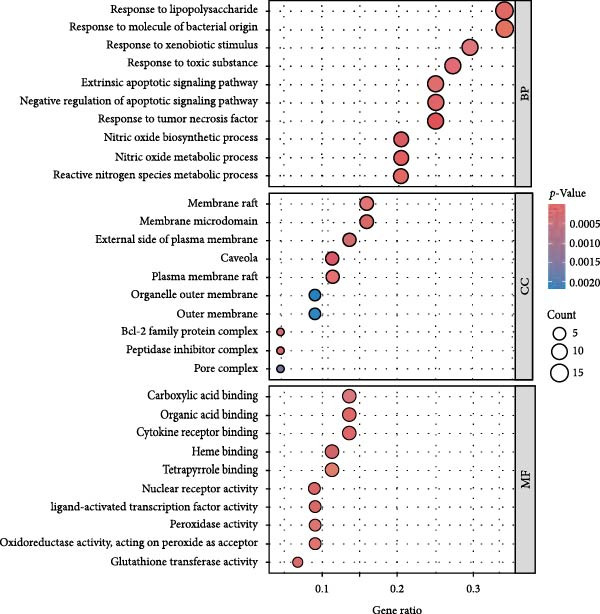
(B)

**Figure:**
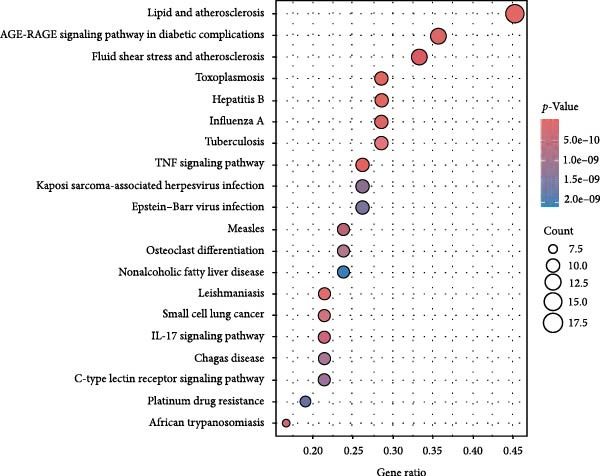
(C)

**Figure:**
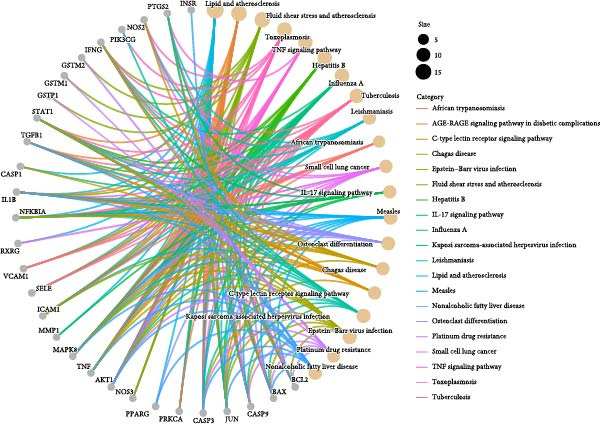
(D)

### 3.4. PPI Network Construction and Identification of Core Targets

**Figure 6:**
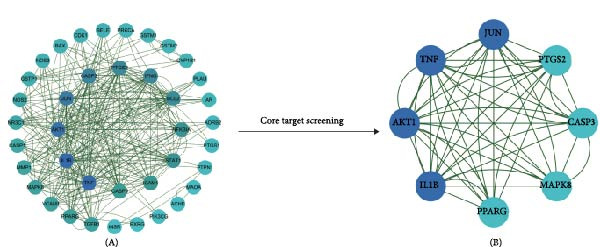
PPI network analysis of molecular targets using core target screening. (A) The initial network consists of numerous interconnected nodes representing potential targets, with edges signifying their interactions. (B) Core target screening identifies and highlights a subset of key targets in darker blue. This subset represents the most critical targets in the network, including TNF, JUN, AKT1, IL1B, CASP3, PPARG, MAPK8, and PTGS2, which are central to the network’s biological processes and are likely crucial in the studied context.

We conducted a PPI network analysis to explore the intrinsic relationships and key targets between ginseng and DR cotargets. By importing the ginseng‐DR cotargets into STRING and further visualization via Cytoscape, we constructed a high‐confidence PPI network with a confidence score of ≥ 0.700 (Figure 6A). To identify the core targets, we first calculated key topological parameters of the PPI network, including BC, CC, and DC, using the Cytoscape application “CytoNCA” (Table 2). Subsequently, we employed CentiScaPe, a Cytoscape application designed for calculating centrality indices, to identify the most crucial nodes in the network. Targets with BC > 40.153, CC > 0.063, and DC > 15.693 were considered core targets, resulting in a total of eight key targets, including Jun proto‐oncogene (JUN), TNF, AKT serine/threonine kinase 1 (AKT1), interleukin 1 beta (IL1B), peroxisome proliferator‐activated receptor gamma (PPARG), mitogen‐activated protein kinase 8 (MAPK8), caspase 3 (CASP3), and prostaglandin‐endoperoxide synthase 2 (PTGS2) (Figure 6B).

**Table 2:** Topological parameters of eight key targets.

Target	Degree	Betweenness	Closeness
TNF	44	157.86246	0.28787878
IL1B	44	261.3579	0.29007635
AKT1	40	182.54535	0.2835821
JUN	36	235.78499	0.2857143
CASP3	32	60.06526	0.2733813
PTGS2	30	45.81468	0.2733813
MAPK8	18	75.11154	0.26573426
PPARG	18	84.5686	0.26206896

### 3.5. Molecular Docking Results Between Core Targets and Active Ingredients in Ginseng

**Figure 7:**
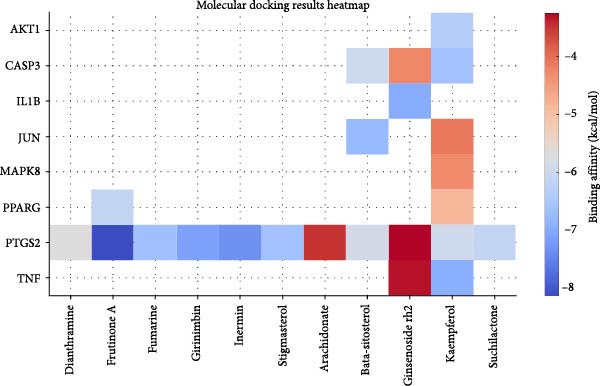
Heatmap of molecular docking results between ginseng active compounds and key target proteins.

To investigate potential drug‐target interactions, we performed molecular docking analysis to predict the binding affinities between the core targets and active compounds. We selected eight core targets as receptors, while the interacting compounds from ginseng were used as ligands. A lower binding affinity score indicates stronger binding activity between the compound and the target. The docking scores for three target‐compound combinations were ≤ −7 kcal/mol, indicating robust binding interactions (Table 3 and Figure 7). The molecular docking results were further visualized using PyMol software to generate corresponding images (Figure 8).

Figure 8Molecular docking results. (A) PTGS2 and Frutinone A (−8.14 kcal/mol). (B) PTGS2 and Girinimbin (−7.1 kcal/mol). (C) PTGS2 and Inermin (−7.32 kcal/mol).

**Figure:**
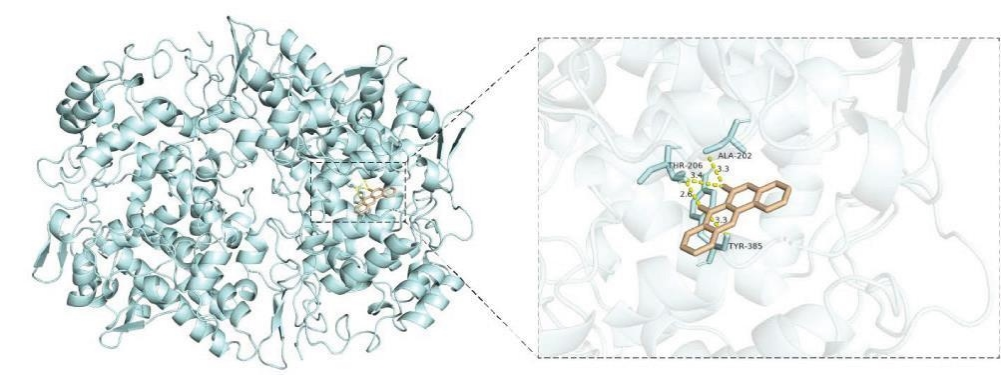
(A)

**Figure:**
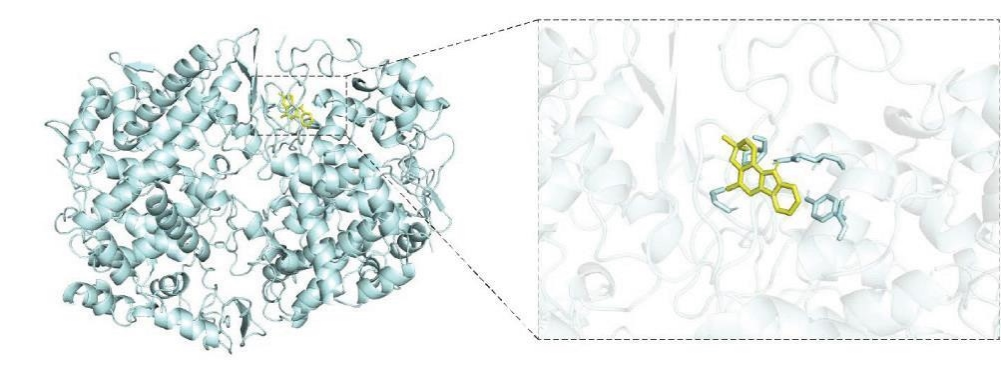
(B)

**Figure:**
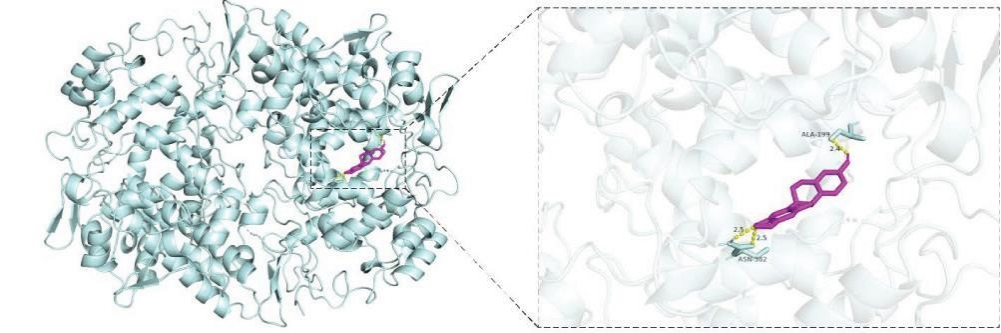
(C)

**Table 3:** Molecular docking results.

Proteins	PDB ID	Test compounds	Affinity (kcal/mol)
TNF	1A8M	Kaempferol	−6.93
		Ginsenoside rh2	−3.31

IL1B	1HIB	Ginsenoside rh2	−6.97

AKT1	1UNP	Kaempferol	−6.36

JUN	1A02	Beta‐sitosterol	−6.76
		Kaempferol	−4.1

CASP3	1CP3	Kaempferol	−6.61
		Ginsenoside rh2	−4.27
		Beta‐sitosterol	−6.01

PTGS2	5F19	Dianthramine	−5.69
		Arachidonate	−3.48
		Frutinone A	−8.14
		Ginsenoside rh2	−3.25
		Girinimbin	−7.1
		Suchilactone	−6.14
		Stigmasterol	−6.61
		Fumarine	−6.62
		Beta‐sitosterol	−5.89
		Inermin	−7.32
		Kaempferol	−5.96

MAPK8	2GMX	Kaempferol	−4.31

PPARG	1FM6	Frutinone A	−6.12
		Kaempferol	−4.89

### 3.6. MD Simulation Results

**Figure 10:**
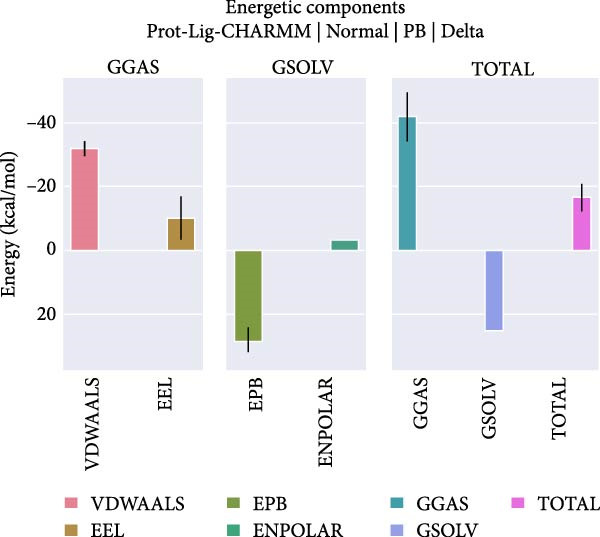
The MMPBSA binding free energy of −16.7 kcal/mol reflects strong, stable protein–ligand interactions.

PTGS2 and Frutinone A are the group with the lowest binding energy in molecular docking. To further verify the interaction between the two, we performed MD simulation analysis. Root mean square deviation (RMSD) assesses structural similarity across molecular conformations during simulations by comparing atomic positions to a reference structure. As shown in Figure 9A, the small molecule exhibited minimal fluctuation (ΔRMSD < 0.2 nm), indicating early stabilization and negligible conformational changes—likely a result of effective energy minimization. Root mean square fluctuation (RMSF) quantifies the flexibility of individual residues. Higher values indicate greater atomic displacement, often linked to dynamic regions. As seen in Figure 9B, residues at the N‐ and C‐termini showed higher fluctuations due to exposure to solvent and lack of structural constraints. In contrast, residues 102–158, 190−272, and 290–405 were more stable, while intermediate regions displayed flexibility, potentially reflecting dynamic binding interfaces or intrachain interactions. The radius of gyration (*R*_g_) measures the compactness of the protein structure. As shown in Figure 9C, *R*_g_ remained stable (∆*R*_g_ < 0.2 nm) throughout the simulation, suggesting overall structural integrity. Minor fluctuations may reflect local domain motions (e.g., bending of α‐helices or expansion of β‐sheets), supporting a design of rigid cores and flexible domains essential for biological function. Hydrogen bonding plays a vital role in molecular recognition and stability. During the simulation, the ligand consistently formed 2–3 hydrogen bonds with the protein (Figure 9D), alongside 3–4 other nonhydrogen interactions, occasionally increasing to 7. This network of interactions supports strong and stable ligand binding. Based on the RMSD stability, MMPBSA was applied to estimate the binding free energy. The total energy was −16.7 kcal/mol (Figure 10), indicating strong noncovalent interactions, particularly electrostatic and van der Waals forces, that stabilized the ligand within the binding pocket and limited conformational drift.

Figure 9Molecular dynamics analysis of the protein–ligand complex. (A) RMSD of the ligand showing stable conformation (ΔRMSD < 0.2 nm). (B) RMSF of protein residues, with higher flexibility at termini and stable core regions. (C) Radius of gyration (*R*_g_) indicating overall structural stability (Δ*R*_g_ < 0.2 nm). (D) Hydrogen bonds and other close‐range interactions between protein and ligand remained consistently stable.

**Figure:**
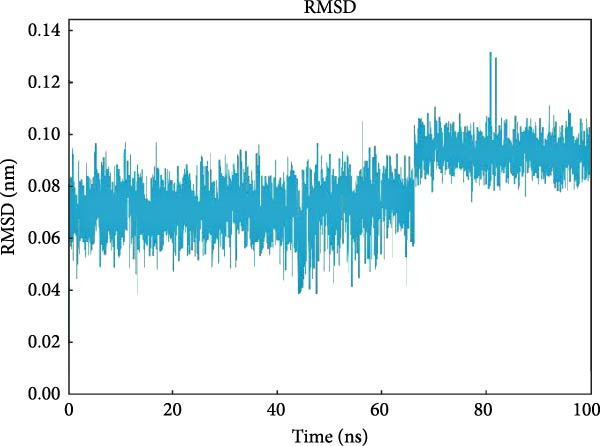
(A)

**Figure:**
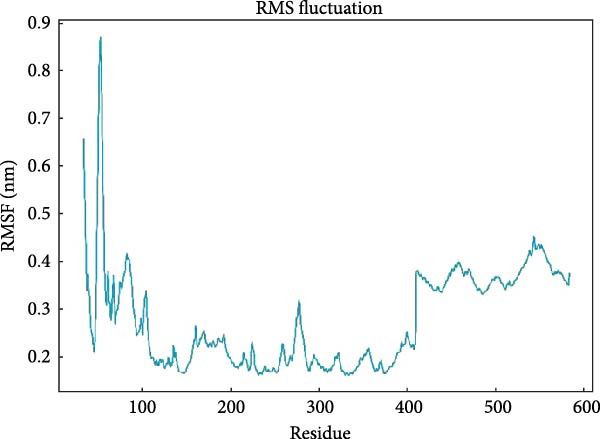
(B)

**Figure:**
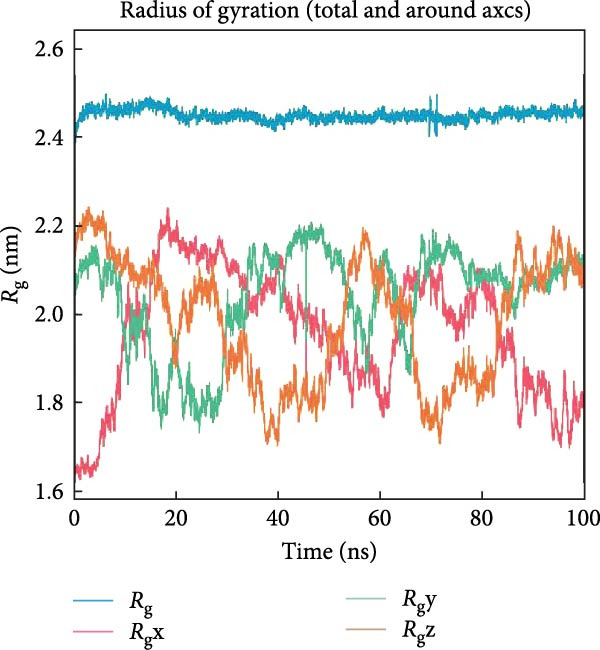
(C)

**Figure:**
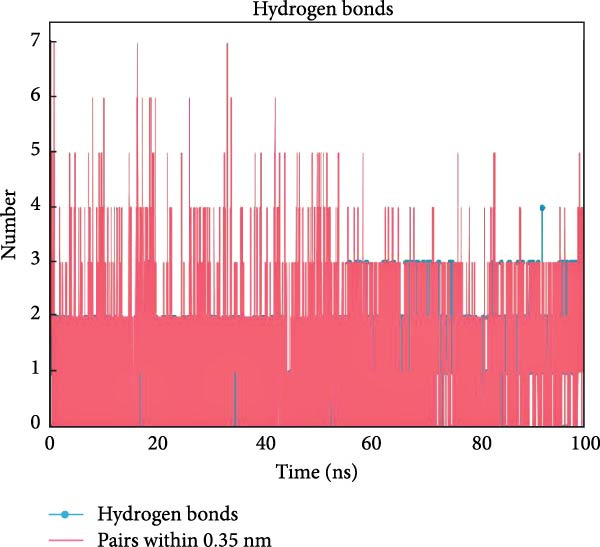
(D)

## 4. Discussion

DR is still a leading cause of adult blindness. Current treatment strategies, such as anti‐VEGF antibodies, primarily target the late stages of the disease, including diabetic macular edema and PDR. However, these treatments do not address neuronal degeneration and, as a result, have limited effectiveness in restoring vision. This highlights an urgent need for new therapeutic approaches focusing on early intervention to delay DR progression and reduce neuronal degeneration [7, 28]. Previous studies have demonstrated that ginseng treatment significantly prevents oxidative stress and the upregulation of extracellular matrix proteins and vasoactive factors induced by diabetes in the retina and heart of diabetic mouse models, suggesting ginseng may delay the development of DR and diabetic cardiomyopathy [13]. Due to the diverse range of phytochemicals present in ginseng, it exhibits multiple pharmacological effects [29]. However, no research currently specifically explores ginseng’s mechanisms in treating DR. In this research, we employed a network pharmacology approach to comprehensively analyze the therapeutic mechanisms of ginseng in DR. Given that DR is a multifactorial disease and ginseng exerts effects through multiple targets, network pharmacology provides a systematic framework to elucidate its mechanisms from a multicomponent, multitarget, and multipathway perspective, while also taking into account the complex pathogenesis of DR.

This study showed that the 44 common molecular targets were significantly enriched in the KEGG pathways, particularly along the AGE‐RAGE signaling pathway, which plays a vital role in the pathogenesis of diabetic complications [30, 31]. In the pathogenesis of DR, AGEs play a dual role. They can directly crosslink with extracellular matrix proteins, altering the structure of microvessels, and they can also bind to their receptor, RAGE. This binding disrupts cellular antioxidant defenses and promotes the production of reactive oxygen species (ROS), leading to irreversible damage to retinal capillaries and vascular tissues [32, 33]. In this study, we identified that ginseng exerts its effects on downstream signaling molecules involved in the AGE‐RAGE pathway, including Akt, TNF, JNK, Bcl‐2, CASP3, IL‐1, and ICAM‐1, among others. Such simultaneous regulation of oxidative stress, inflammation, and apoptosis underscores the multicomponent and multitarget nature of ginseng. Moreover, given that DR progression involves the interplay of vascular dysfunction, neurodegeneration, and chronic inflammation, the ability of ginseng to act on multiple signaling axes provides a mechanistic rationale for its therapeutic potential in delaying or preventing retinal damage.

We also constructed a PPI network for 44 molecular targets that respond to ginseng and DR, identifying 8 of these as core targets. While these targets have not been directly reported in other studies on ginseng therapy for DR, some have been indirectly validated through related targets within the same pathways. These eight core targets are crucial in inducing inflammation, promoting cell apoptosis, and regulating metabolism. IL‐1β is a pro‐inflammatory cytokine secreted by monocytes and macrophages [34]. The role of IL‐1β in the progression of DR has been extensively studied. Elevated protein levels of IL‐1β have been observed in DR patients’ vitreous and aqueous humor. Rodent studies further support these findings, showing that IL‐1β is upregulated following streptozotocin (STZ)‐induced diabetes. However, administration of anti‐inflammatory or antioxidant drugs has been found to inhibit this upregulation and significantly reduce IL‐1β levels, suggesting a potential therapeutic target for DR management [35, 36]. AKT, also known as protein kinase B (PKB), is a crucial enzyme in cellular signaling pathways, regulating metabolism, cell survival, growth, and proliferation [37]. In insulin signaling, AKT is activated by phosphoinositide 3‐kinase (PI3K), leading to the translocation of glucose transporter type 4 (GLUT4) to the cell surface and promoting glucose uptake. Research indicates that AKT signaling is impaired in DR [38, 39]. Studies have shown reduced AKT and phosphorylated AKT (p‐AKT) levels in the retinas of diabetes‐induced rat models, particularly those subjected to a high‐fat diet [40]. This evidence suggests that ginseng has potential as a treatment for DR, but it also highlights the need for further exploration into the specific molecular mechanisms involved.

Through molecular docking and MD simulation analysis, we found that PTGS2 exhibited a strong binding affinity with various active ingredients of ginseng. This suggests that PTGS2 is likely to be one of the effective targets for ginseng in the treatment of DR. Research has shown that PTGS2, a well‐known inflammatory marker, is upregulated in DR and plays a role in promoting inflammation [41]. Additionally, PTGS2, being an iron‐related protein, contributes to the progression of DR by promoting ferroptosis (iron‐dependent cell death) in retinal cells [42, 43]. Some network pharmacology studies have also suggested that other traditional Chinese medicines exert protective effects against DR by targeting PTGS2, further emphasizing their potential as therapeutic targets [44, 45]. Therefore, further experimental studies are advised to investigate the specific mechanisms by which ginseng targets PTGS2 to protect against DR.

## 5. Conclusion

In conclusion, this study highlights the potential therapeutic benefits of ginseng in treating DR. We identified 22 active compounds in ginseng that affect the development and progression of DR by interacting with at least 44 potential target proteins. These targets are notably enriched in the AGE‐RAGE signaling pathway, which is closely linked to diabetes‐related complications. Furthermore, we constructed a PPI network and identified eight core targets, including JUN, TNF, AKT1, IL1B, PPARG, MAPK8, CASP3, and PTGS2. Future studies will aim to validate these findings through additional in vitro and in vivo experiments. Overall, this research offers a new theoretical framework for using ginseng in the treatment of DR.

## Disclosure

All authors have approved the final manuscript.

## Conflicts of Interest

The authors declare no conflicts of interest.

## Author Contributions

Yuanchao Fu and Chengzhi Liu designed and implemented the experiments and wrote and revised the manuscript. Chuanlie Zhou and Guzhi Xu collected the data.

## Funding

This work was supported by the Dalian Traditional Chinese Medicine Scientific Research Plan Project (23Z11025).

## Supporting Information

Additional supporting information can be found online in the Supporting Information section.

## Supporting information


**Supporting Information 1** Table S1: List of the 22 active ingredients and their corresponding 117 related targets.

**Supporting Information 2** Table S2: A list of 3894 unique DR‐related targets obtained in this study.

## Data Availability

The data that support the findings of this study are available from the corresponding author upon reasonable request.

## References

[1] Wong T. Y. , Cheung C. M. G. , Larsen M. , Sharma S. , and Simó R. , Diabetic Retinopathy, Nature Reviews Disease Primers. (2016) 2, no. 1, 10.1038/nrdp.2016.12, 2-s2.0-85006701476, 16012. 27159554

[2] Cheung N. , Mitchell P. , and Wong T. Y. , Diabetic Retinopathy, The Lancet. (2010) 376, no. 9735, 124–136, 10.1016/S0140-6736(09)62124-3, 2-s2.0-77955013602. 20580421

[3] Teo Z. L. , Tham Y. C. , and Yu M. , et al.Global Prevalence of Diabetic Retinopathy and Projection of Burden Through 2045: Systematic Review and Meta-Analysis, Ophthalmology. (2021) 128, no. 11, 1580–1591, 10.1016/j.ophtha.2021.04.027. 33940045

[4] Amin J. , Sharif M. , and Yasmin M. , A Review on Recent Developments for Detection of Diabetic Retinopathy, Scientifica (Cairo). (2016) 2016, 20, 10.1155/2016/6838976, 2-s2.0-85018937113, 6838976. 27777811 PMC5061953

[5] Yau J. W. , Rogers S. L. , and Kawasaki R. , et al.Global Prevalence and Major Risk Factors of Diabetic Retinopathy, Diabetes Care. (2012) 35, no. 3, 556–564, 10.2337/dc11-1909, 2-s2.0-84859030420. 22301125 PMC3322721

[6] Stewart M. W. , Treatment of Diabetic Retinopathy: Recent Advances and Unresolved Challenges, World Journal of Diabetes. (2016) 7, no. 16, 333–341, 10.4239/wjd.v7.i16.333. 27625747 PMC4999649

[7] Zafar S. , Sachdeva M. , Frankfort B. J. , and Channa R. , Retinal Neurodegeneration as an Early Manifestation of Diabetic Eye Disease and Potential Neuroprotective Therapies, Current Diabetes Reports. (2019) 19, no. 4, 10.1007/s11892-019-1134-5, 2-s2.0-85062076382. PMC7192364 30806815

[8] Barber A. J. and Baccouche B. , Neurodegeneration in Diabetic Retinopathy: Potential for Novel Therapies, Vision Research. (2017) 139, 82–92, 10.1016/j.visres.2017.06.014, 2-s2.0-85031427153. 28988945

[9] Mancuso C. and Santangelo R. , *Panax ginseng* and *Panax quinquefolius*: From Pharmacology to Toxicology, Food and Chemical Toxicology. (2017) 107, 362–372, 10.1016/j.fct.2017.07.019, 2-s2.0-85023638775. 28698154 PMC7116968

[10] Kiefer D. and Pantuso T. , Panax ginseng, American Family Physician. (2003) 68, no. 8, 1539–1542. 14596440

[11] Ito H. and Ito M. , Recent Trends in Ginseng Research, Journal of Natural Medicines. (2024) 78, no. 3, 455–466, 10.1007/s11418-024-01792-4. 38512649

[12] Li K. , Wang Y. J. , Chen C. , Wang X. J. , and Li W. , Targeting Pyroptosis: A Novel Strategy of Ginseng for the Treatment of Diabetes and Its Chronic Complications, Phytomedicine. (2025) 138, 10.1016/j.phymed.2025.156430, 156430. 39892311

[13] Sen S. , Chen S. , Wu Y. , Feng B. , Lui E. K. , and Chakrabarti S. , Preventive Effects of North American Ginseng (*Panax quinquefolius*) on Diabetic Retinopathy and Cardiomyopathy, Phytotherapy Research. (2013) 27, no. 2, 290–298, 10.1002/ptr.4719, 2-s2.0-84873405900. 22566158

[14] Wan Y. , Liu D. , and Xia J. , et al.Ginsenoside CK, Rather Than Rb1, Possesses Potential Chemopreventive Activities in Human Gastric Cancer via Regulating PI3K/AKT/NF-κB Signal Pathway, Frontiers in Pharmacology. (2022) 13, 10.3389/fphar.2022.977539, 977539. 36249752 PMC9556731

[15] Guo L. , Zhen Q. , and Zhen X. , et al.A Network Pharmacology Approach to Explore and Validate the Potential Targets of Ginsenoside on Osteoporosis, International Journal of Immunopathology and Pharmacology. (2022) 36, 10.1177/03946320221107239, 3946320221107239. 35791093 PMC9272184

[16] Zhang P. , Zhang D. , and Zhou W. , et al.Network Pharmacology: Towards the Artificial Intelligence-Based Precis Ion Traditional Chinese Medicine, Briefings in Bioinformatics. (2024) 25, no. 1, 10.1093/bib/bbad518, bbad518. PMC10777171 38197310

[17] Ru J. , Li P. , and Wang J. , et al.TCMSP: A Database of Systems Pharmacology for Drug Discovery From Herb Al Medicines, Journal of Cheminformatics. (2014) 6, no. 1, 10.1186/1758-2946-6-13, 2-s2.0-84899981355. PMC4001360 24735618

[18] Barrett T. , Wilhite S. E. , and Ledoux P. , et al.NCBI GEO: Archive for Functional Genomics Data Sets--Update, Nucleic Acids Research. (2012) 41, no. D1, D991–D995, 10.1093/nar/gks1193, 2-s2.0-84874271270. 23193258 PMC3531084

[19] Võsa U. , Claringbould A. , and Westra H. J. , et al.Large-Scale Cis- and Trans-eQTL Analyses Identify Thousands of Genetic Loci and Polygenic Scores that Regulate Blood Gene Expression, Nature Genetics. (2021) 53, 1300–1310. 34475573 10.1038/s41588-021-00913-z PMC8432599

[20] Zhu Z. , Zhang F. , and Hu H. , et al.Integration of Summary Data From GWAS and eQTL Studies Predicts Complex Trait Gene Targets, Nature Genetics. (2016) 48, no. 5, 481–487, 10.1038/ng.3538, 2-s2.0-84961927084. 27019110

[21] Safran M. , Rosen N. , and Twik M. , et al. Abugessaisa I. and Kasukawa T. , The GeneCards Suite., Practical Guide to Life Science Databases, 2021, Springer.

[22] Amberger J. S. and Hamosh A. , Searching Online Mendelian Inheritance in Man (OMIM): A Knowledgebase of Human Genes and Genetic Phenotypes, Current Protocols in Bioinformatics. (2017) 58, no. 1, 1.2.1–1.2.12, 10.1002/cpbi.27, 2-s2.0-85029433513. PMC5662200 28654725

[23] Zhou Y. , Zhang Y. , and Lian X. , et al.Therapeutic Target Database Update 2022: Facilitating Drug Discovery With Enriched Comparative Data of Targeted Agents, Nucleic Acids Research. (2022) 50, no. D1, D1398–D1407, 10.1093/nar/gkab953. 34718717 PMC8728281

[24] Chen L. , Zhang Y. H. , Wang S. , Zhang Y. , Huang T. , and Cai Y. D. , Prediction and Analysis of Essential Genes Using the Enrichments of Gene Ontology and KEGG Pathways, PLOS ONE. (2017) 12, no. 9, 10.1371/journal.pone.0184129, 2-s2.0-85028940999. PMC5584762 28873455

[25] Halperin I. , Ma B. , Wolfson H. , and Nussinov R. , Principles of Docking: An Overview of Search Algorithms and a Guide to Scoring Functions, Proteins: Structure, Function, and Bioinformatics. (2002) 47, no. 4, 409–443, 10.1002/prot.10115, 2-s2.0-0036606483. 12001221

[26] Eberhardt J. , Santos-Martins D. , Tillack A. F. , and Forli S. , AutoDock Vina 1.2.0: New Docking Methods, Expanded Force Field, and Python Bindings, Journal of Chemical Information and Modeling. (2021) 61, no. 8, 3891–3898, 10.1021/acs.jcim.1c00203. 34278794 PMC10683950

[27] Seeliger D. and de Groot B. L. , Ligand Docking and Binding Site Analysis with PyMOL and Autodock/Vina, Journal of Computer-Aided Molecular Design. (2010) 24, no. 5, 417–422, 10.1007/s10822-010-9352-6, 2-s2.0-77953325845. 20401516 PMC2881210

[28] Hammes H.-P. , Diabetic Retinopathy: Hyperglycaemia, Oxidative Stress and beyond, Diabetologia. (2018) 61, no. 1, 29–38, 10.1007/s00125-017-4435-8, 2-s2.0-85029770762. 28942458

[29] Yin X. , Hu H. , Shen X. , Li X. , Pei J. , and Xu J. , Ginseng Omics for Ginsenoside Biosynthesis, Current Pharmaceutical Biotechnology. (2021) 22, no. 5, 570–578, 10.2174/1389201021666200807113723. 32767915

[30] Xu J. , Chen L. J. , and Yu J. , Involvement of Advanced Glycation End Products in the Pathogenesis of Diabetic Retinopathy, Cellular Physiology and Biochemistry. (2018) 48, no. 2, 705–717, 10.1159/000491897, 2-s2.0-85051092679. 30025404

[31] Vlassara H. , The AGE-Receptor in the Pathogenesis of Diabetic Complications, Diabetes/Metabolism Research and Reviews. (2001) 17, no. 6, 436–443, 10.1002/dmrr.233, 2-s2.0-0035668462. 11757079

[32] Kandarakis S. A. , Piperi C. , Moschonas D. P. , Korkolopoulou P. , Papalois A. , and Papavassiliou A. G. , Dietary Glycotoxins Induce RAGE and VEGF up-Regulation in the Retina of Normal Rats, Experimental Eye Research. (2015) 137, 1–10, 10.1016/j.exer.2015.05.017, 2-s2.0-84931265286. 26026876

[33] Yamagishi S. , Role of Advanced Glycation End Products (AGEs) and Receptor for AGEs (RAGE) in Vascular Damage in Diabetes, Experimental Gerontology. (2011) 46, no. 4, 217–224, 10.1016/j.exger.2010.11.007, 2-s2.0-79952707168. 21111800

[34] Lopez-Castejon G. and Brough D. , Understanding the Mechanism of IL-1β Secretion, Cytokine and Growth Factor Reviews. (2011) 22, no. 4, 189–195, 10.1016/j.cytogfr.2011.10.001, 2-s2.0-81155134753. 22019906 PMC3714593

[35] Wooff Y. , Man S. M. , Aggio-Bruce R. , Natoli R. , and Fernando N. , IL-1 Family Members Mediate Cell Death, Inflammation and Angiogenesis in Retinal Degenerative Diseases, Frontiers in Immunology. (2019) 10, 10.3389/fimmu.2019.01618, 2-s2.0-85069500027. PMC6646526 31379825

[36] Natoli R. , Fernando N. , and Madigan M. , et al.Microglia-Derived IL-1β Promotes Chemokine Expression by Müller Cells and RPE in Focal Retinal Degeneration, Molecular Neurodegeneration. (2017) 12, no. 1, 10.1186/s13024-017-0175-y, 2-s2.0-85018585360. PMC5404662 28438165

[37] Manning B. D. and Toker A. , AKT/PKB Signaling: Navigating the Network, Cell. (2017) 169, no. 3, 381–405, 10.1016/j.cell.2017.04.001, 2-s2.0-85018509261. 28431241 PMC5546324

[38] Liu C. , Dong W. , Li J. , Kong Y. , and Ren X. , O-GlcNAc Modification and Its Role in Diabetic Retinopathy, Metabolites. (2022) 12, no. 8, 10.3390/metabo12080725. PMC9415332 36005597

[39] Li Y. , Liu J. , Ma X. , and Bai X. , Maresin-1 Inhibits High Glucose Induced Ferroptosis in ARPE-19 Cells by Activating the Nrf2/HO-1/GPX4 Pathway, BMC Ophthalmology. (2023) 23, no. 1, 10.1186/s12886-023-03115-9. PMC10481498 37674121

[40] Marçal A. C. , Leonelli M. , and Fiamoncini J. , et al.Diet-Induced Obesity Impairs AKT Signalling in the Retina and Causes retinal Degeneration, Cell Biochemistry and Function. (2013) 31, no. 1, 65–74, 10.1002/cbf.2861, 2-s2.0-84872387225. 22915345

[41] Chen Z. , Liu B. , and Zhou D. , et al.AQP4 Regulates Ferroptosis and Oxidative Stress of Muller Cells in Diabetic Retinopathy by Regulating TRPV4, Experimental Cell Research. (2024) 439, no. 1, 10.1016/j.yexcr.2024.114087, 114087. 38735619

[42] Huang Y. , Peng J. , and Liang Q. , Identification of Key Ferroptosis Genes in Diabetic Retinopathy Based on Bioinformatics Analysis, PLOS ONE. (2023) 18, no. 1, 10.1371/journal.pone.0280548, e0280548. 36689408 PMC9870164

[43] Li J. , Chen K. , and Li X. , et al.Mechanistic Insights Into the Alterations and Regulation of the AKT Signaling Pathway in Diabetic Retinopathy, Cell Death Discovery. (2023) 9, no. 1, 10.1038/s41420-023-01717-2. PMC10656479 37978169

[44] Wang Y. , Xue B. , Wang X. , Wang Q. , Liu E. , and Chen X. , Pharmacokinetic Study of Tangwang Mingmu Granule for the Management of Diabetic Retinopathy Based on Network Pharmacology, Pharmaceutical Biology. (2021) 59, no. 1, 1332–1348, 10.1080/13880209.2021.1979051. PMC8491704 34590544

[45] Lin X. , Bao M. , and Zhang X. , et al.Study on the Bioactive Ingredients and Mechanism of Huangqi Against Diabetic Retinopathy Based on Network Pharmacology and Experimental Verification, Journal of the Chinese Medical Association. (2024) 87, no. 8, 789–798, 10.1097/JCMA.0000000000001113. 38780966 PMC13610169

